# Natriuretic Peptides in Kawasaki Disease: the Myocardial Perspective

**DOI:** 10.3390/diagnostics3010001

**Published:** 2013-01-10

**Authors:** Nagib Dahdah, Anne Fournier

**Affiliations:** Division of Pediatric Cardiology, CHU Ste-Justine, University of Montreal, Montreal, QC H3T 1C5, Canada

**Keywords:** Kawasaki disease, myocarditis, NT-proBNP, hyponatremia, diagnosis, prognosis

## Abstract

Making a diagnosis of Kawasaki disease with certainty may be challenging, especially since the recognition of cases with incomplete diagnostic criteria and its consequences. In order to build the diagnostic case in daily practice, clinicians rely on clinical criteria established over four decades ago, aided by non specific laboratory tests, and above all inspired by experience. We have recently studied the diagnostic value of N-terminal pro B-type natriuretic peptide to improve the diagnostic certainty of cases with complete or incomplete clinical criteria. Our working hypothesis was based on the fact that myocarditis is present in nearly all Kawasaki disease patients supported by histology data. In this paper, we review these facts and the myocardial perspective from the diagnostic and the mechanistic standpoints.

## 1. Introduction

Kawasaki disease (KD) is a febrile illness of childhood [1]. It is caused by a transient dysregulation of the immune system, likely triggered by a common infection in genetically susceptible patients [2,3]. It causes vasculitis of large and medium vessels [4]. Coronary artery (CA) vasculitis is the most significant complication of KD [5], which is the main focus of research and recommendations for treatment and follow-up. Above all, KD consistently causes myocarditis, which paved our way towards new working hypotheses. In this review, we will discuss the disease, the diagnostic challenge, and the emerging trend from the myocardial perspective.

## 2. Review

### 2.1. History and Epidemiological Trend of KD

KD was first described in Japan in 1967 [6]. It reached a global perspective in the mid 1970s following increasing reports from Western countries [7,8,9]. The disease typically involves preschool children, but can occur in older children [10]. Although a “self limiting disease”, a troubling number of children succumbed in the early years to cardiac complications [11] until intravenous immunoglobulin (IVIG) therapy proved to be protective and beneficial in the 1980’s [12], then optimized in the 1990’s [13]. Left untreated, patients are at increased risk of CA aneurismal deformation, which favors intracoronary thrombosis [14], and CA scarring with heavy calcification and stenosis [15], with subsequent myocardial infarction and death [16]. Current efforts, including therapeutic trials involving tumor necrosis factor blockers [17,18], aim at reducing such consequences. Nevertheless, the diagnosis of KD has long been largely based on the originally reported clinical criteria from over four decades ago [6]. It is well known however that a substantial number of patients may be missed due to the non specificity of these criteria [19,20]. A recent US study displayed an impressive increase of the incidence of KD with incomplete criteria (iKD) from 24% between 2000 and 2002 to 47% between 2007 and 2009 [21]. In addition, the biological markers clinicians rely upon to support the diagnosis of KD (white blood cells, platelet count, hematocrit, C-reactive protein, erythrocyte sedimentation rate, liver enzymes, serum albumin, *etc.*) are not specific for the disease [22]. Early recommendations suggested that an infectious origin of the symptoms must be ruled out in order to support the diagnosis of KD, but this also has been solidly discredited [10]. Currently, with four or five clinical criteria in addition to fever (Table 1), patients are labeled as cases with complete KD criteria (cKD). Otherwise, they are sometimes referred to as atypical KD, or more appropriately with the preferred designation of iKD. Patients with iKD are at considerable risk for CA complications [23]. In 2004 and 2005, the American [19] and the Japanese [24] medical authorities recommended broader diagnostic definition of the disease for the inclusion of iKD. Although these recommendations actually increased the awareness of diagnosing iKD in the absence of a gold standard, the likelihood of wrongfully diagnosing (over-diagnosing, or under-diagnosing) children with KD became at stake, which triggered our interest in refining the diagnostic certainty of KD. 

**Table 1:** List of the classical clinical criteria for the diagnosis of Kawasaki disease.

1) Fever for five days or more
2) Bilateral conjunctival injection without exudate
3) Polymorphous exanthem (skin rash)
4) Changes in lips and mouth (mucositis or enanthema): Reddened, dry, or cracked lips Strawberry tongue Diffuse redness of oral or pharyngeal mucosa
5) Changes in extremities: Reddening of palms or soles Firm oedema of hands or feet Desquamation of skin of hands, feet, and groin (convalescent phase)
6) Cervical lymphadenopathy: More than 15 mm in diameter usually unilateral, single, non-purulent, and painful

### 2.2. The Myocardium

In a search for a reliable target, we focused on the earliest and the commonest KD pathology feature, the myocarditis. We also tried to identify the most reliable diagnostic method to reflect the pathophysiological myocardial alteration in acute KD. The diagnostic tool had to be readily available, operator independent and financially sound. It was essential that the diagnosis be made in the optimal therapeutic window of 10 days since onset of fever, after which the likelihood of response to IVIG therapy becomes rather unlikely [25]. The answer to the first question was in the early post mortem pathology reports where it was well described that myocarditis was the earliest feature, overwhelmingly present in the first nine days of the illness [26,27]. Indeed, the post mortem cardiac pathology features classified KD into four clinicopathological stages. Stage 1 (0 to 9 days of illness) is characterized by the presence of carditis involving the three layers of the heart with evidence of angiitis of the major, minor and microscopic coronary vessels; Stage II (12 to 25 days) inflammation and aneurysm with thrombus of the major CA are present in addition to pancarditis, including the conduction system; Stage III (28 to 31 days) represented the healing process with granulation of the main CA and disappearance of inflammation in the microvessels; Stage IV (beyond 40 days of illness) is characterized by sequelae involving CA stenoses in addition to myocardial fibrosis, old pericarditis and focal endocardial fibroelastosis [26]. Although the incidence of clinical myocarditis remains difficult to estimate in KD, there are available data beyond the post mortem reports. Live biopsy series from 201 patients at various stages of the disease revealed the presence of myocardial inflammatory features in 100% of cases (myocarditis, cellular infiltration and fibrosis) regardless of the presence or absence of significant CA injury [28]. Ultrastructural histology studies also provided further evidence of otherwise unrecognized myocardial damage, both in children with overt CA involvement and in cases with milder involvement (*i.e.*, with regressed CA aneurysms or mild CA dilatation), albeit to a lesser degree in the latter group [29]. The pathology reports were followed by imaging determinants of myocardial infiltrates using nuclear imaging scan detecting labeled white blood cells in the myocardium of 87% of KD patients [30,31]. Similarly, echocardiography parameters suggestive of transitory myocardial edema, systolic and diastolic disturbances and transitory valvular dysfunction confirmed the histology findings from a dynamic and a functional perspective [32,33,34,35]. Although echocardiographic findings were limited to 28% of subjects free of significant CA lesions [32,33], myocarditis is clinically subtle in the vast majority of cases, with a very few cases exhibiting severe clinical presentation [36]. The next challenge is to detect and possibly quantify subclinical myocarditis.

### 2.3. Hyponatremia and the Natriuretic Peptides

**Figure 1:**
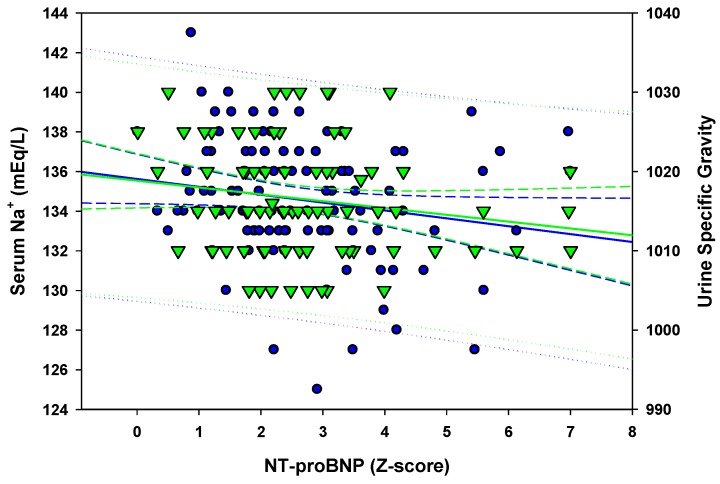
With higher serum NT-proBNP, there is lower serum sodium concentration (blue circles) concomitant with lower urine specific gravity (green triangles). This observation contradicts the theory of an increased antidiuretic hormone activity where an elevated specific gravity is expected with hyponatremia.

A bridge to the next question lies perhaps in the observed low serum Na in acute KD compared to other febrile illnesses. In our series, hyponatremia (Na < 135 mEq/L) was present in 50% of cases [37], *versus* 26% to 69% in other series [38,39,40]. On a different note, some studies suggested an association between the severity of KD and hyponatremia [39,41,42] with little or no evidence of the possible reasons for such an association. In one study hyponatremia was present in 13/50 (26%) patients, among whom 12% of patients or 41% of hyponatremic subjects presented metabolic features supportive of SIADH [39]. In another study, 27/39 (69%) had hyponatremia and 41% of hyponatremic patients had SIADH [40]. In the latter report, hyponatremia persisted after IVIG, despite an improvement in SIADH metabolic profile. In both studies, natriuretic peptides were significantly higher in subjects with hyponatremia [39] or SIADH [40]. Both observations the persistence of hyponatremia and the increase in natriuretic peptide activity raise the question of the potentially complex etiology of hyponatremia in acute KD. In our recent works, we took a different look on the possible causative mechanisms. When natriuretic peptide secretion increases in KD, one expects natriuresis to increase, with a secondary reduction in plasma concentration of Na. This was indeed our observation in a series of 117 KD cases where blood and urine samples were available upon admission prior to intravenous fluid and electrolyte administration (Figure 1). 

**Figure 2:**
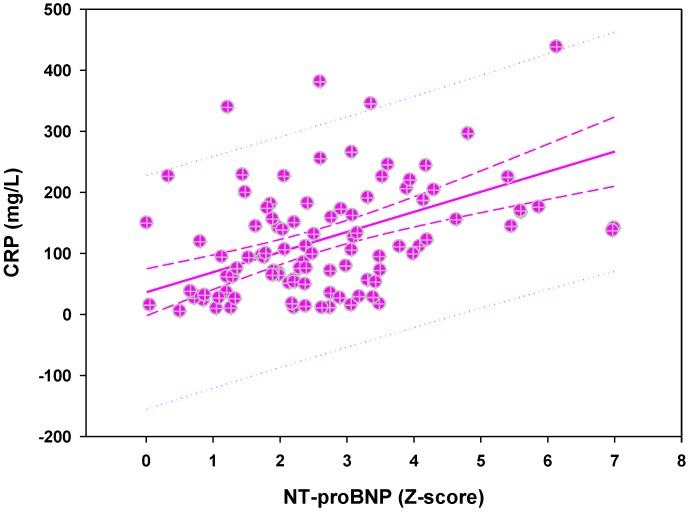
A high serum C-reactive protein (CRP) concentration in association with increased NT-proBNP is a witness of the degree of inflammation.

In parallel, urine specific gravity (USG) was lower with increased NT-proBNP and lower serum sodium, which contradicts the suggested SIADH theory where one expects an increase in USG associated with hyponatremia. Similarly, high serum CRP concentration was associated with elevated NT-proBNP serum concentration (Figure 2). Nevertheless, our use of USG as a surrogate for urine osmolality requires further assessment. Despite our observation, an increase in antiduretic hormone activity may still be possible in some cases as suggested by associated cerebral vasculitis [43,44]. Transitory cerebral perfusion impairment was indeed reported in a series of neurologically asymptomatic KD patients [45]. Finally, renal injury may also represent a potential explanation for sodium loss in KD. Nevertheless, the paucity of focused renal studies does not allow us to draw conclusions at the present time [46,47]. So, is there a role of the heart in the hyponatremia we observe in acute KD?

### 2.4. Towards a New Diagnostic Marker

Fortunately, myocardial infarction post KD is usually limited to cases with severe CA vasculitis [26,48,49,50]. Hence, attempts to measure circulating cardiac enzymes when myocardial infarction was not present were inconclusive. In this perspective, troponin-I was very mildly elevated, below infarction level (0.2–1.2 ng/mL), in a limited series of patients without clinical features of myocarditis. There was no correlation between troponin-I levels and CA dilatation [51]. In another series, 15% had serum levels above those of controls, and were consistent with a concomitant elevation of creatine kinase-MB subunit [52]. The reduction of troponin-I level following a therapeutic response to IVIG, suggested that KD myocarditis and myocardial cytolysis were the cause of elevated serum levels [53]. It is possible that insufficient myocyte damage in acute KD is the limiting factor for the usefulness of an intracellular compartment, such as troponins, in characterizing the subtle myocardial inflammation in acute KD. Elsewhere, it was suggested that the target of myocardial injury in acute KD is probably an inflammatory infiltrate within the extracellular matrix of the myocardium [54,55] rather than the contractile myofilaments [56]. Hence, our search for a biological marker had to represent myocardial inflammation and subsequent myocardial shear stress. 

Natriuretic peptides, such as B-type natruretic peptide (BNP), are natural diuretics normally secreted by the myocardium especially in circumstances with increased intra-cardiac pressure and myocardial stress [57]. PreproBNP, the precursor of BNP, is a 134-amino-acid peptide which, following its release from the cardiomyocytes, is cleaved in the liver during a first pass into a signal peptide and proBNP. ProBNP is then cleaved by a circulating endoprotease into BNP, the active moiety metabolized by the kidney and the vascular tree, and the metabolically inactive NT-proBNP fragment [58]. The clearance is different for those two moieties, BNP’s half-life is 20–30 min short, and NT-proBNP circulates unchanged in the serum for 60–120 min before being cleared primarily by the kidney [59,60]. 

In a search for a biomarker of myocardial distress, we investigated the likelihood of detecting serum concentration of BNP and NT-proBNP in a series of patients with acute KD compared to a series of febrile controls. In a first report, BNP was elevated in cases with complete KD criteria [61], as reported also by another group [62]. However, according to our results, BNP represented no prospects for future developments in iKD. In contrast, we found that NTproBNP was able to discriminate iKD patients from febrile controls in a similar fashion as for cKD patients [61]. The longer half-life of circulating NT-proBNP, its limited physiological fluctuation and its stability in room temperature were probably the reasons of its superiority in a situation where subtle myocarditis was expected. 

Bearing in mind that iKD patients are the most difficult cases to diagnose, our subsequent work focused on NT-proBNP alone [37]. In that study, we compared KD subjects, both cKD and iKD, to children with a variety of febrile illnesses that make part of the list of the usual differential diagnoses [63]. NT-proBNP serum concentration was measured on a commercially available platform using an electrochemiluminescence immunoassay on the Elecsys analyzer (Roche Diagnostics, Indianapolis, IN, USA) with biotinylated polyclonal capture antibodies and polyclonal ruthenium-complexed detection antibodies. Based on previous studies including measurements from pediatric subjects, this methodology offers minimal variation between laboratories (3.8–6.5%), as well as minimal intra- and inter-assay variability (0.9–3.0% and 3.6–5.8%, respectively) [64,65]. The goal of our study was to determine the capability to distinguish KD cases from febrile control subjects. In this perspective, the Receiver Operator Characteristics (ROC) analysis showed the clear advantage of NT-proBNP over the standard biological markers. Because NT-proBNP varies with age during childhood [65,66], a single cut-off value based on the area under the curve from ROC analysis would have been misleading and inappropriate. We hence utilized two additional definitions of abnormal serum levels. One was based on age specific cut-off values [64], and the other was based on the calculation of age-specific Z-scores for NT-proBNP from a log-linear regression model. With these three case definitions of elevated NT-proBNP the odds ratios of separating KD from non KD febrile illnesses varied between 18.13 (95%CI: 7.21–45.57), 20.82 (95%CI: 8.18–53.0), and 26.71 (95%CI: 8.64–82.57) (P < 0.001), respectively. More interestingly, there were no statistically significant differences between cases with cKD and iKD, neither in the ROC analysis nor in the diagnostic power parameters (positive predictive value 82.8–93.4%, negative predictive value 65.2–79.1%, sensitivity 70.4–88.9%, and specificity 69.4–91.8%). This study became the first in four decades to suggest an operator independent biological marker for KD. Although not specific for the disease, it was definitely superior to other laboratory tests.

### 2.5. From Diagnostic Support to Prognostic Potentials

Other groups investigated serum NT-proBNP in acute KD [62,67,68,69,70,71]. According to recent data, NT-proBNP represents a prognostic estimate of KD severity and resistance to IVIG [72,73,74]. In one study, BNP > 50 pg/mL was strongly associated with increased left ventricular mass index as well as with reduced systolic myocardial velocity and contractility [72]. In another study serum NT-proBNP level >1,000 pg/ml was strongly predictive of CA lesions on ROC analysis, followed by hyponatremia and hypoalobuminemia [73]. Accordingly, 6 of 43 patients, all appropriately treated with IVIG, presented CA lesions and had a significantly higher serum NT-proBNP level (1,073 ± 1,427 ng/mL) compared to those without CA lesions (2,611 ± 1,699 ng/ml), in spite of comparable day of illness at diagnosis, C-reactive protein and albumin levels and other standard biological markers. As novel adjunctive therapeutic interventions are under investigation [17,75,76], enhancing risk classification may become instrumental in selecting cases at risk for IVIG resistance [77,78,79]. To that effect, NT-proBNP was recently found to be an independent predictor of resistance to IVIG therapy [74]. In essence, in this series, the 22 IVIG resistant patients had elevated NT-proBNP levels at onset which persisted to a higher extent 48 to 72 h later compared to the 107 responders. Other biological factors, such as neutrophil count and serum albumin, but not initial CRP level, were found to be associated with IVIG resistance in the same series. Nevertheless, the highest odd ratios that were predictive of IVIG resistance were neutrophil count (22.32; 95%CI: 1.88–264.94) followed by NT-proBNP (14.43; 95%CI: 1.27–163.34) then CRP (8.68; 95%CI: 1.14–65.75). Finally, from a cardiovascular standpoint, follow-up recommendations for KD are solely based on the severity and the extent of CA lesions [80]. From our perspective, the KD myocardial injury merits specific considerations. Perhaps characterizing KD myocardial injury with natriuretic peptide release will provide additional insight into remote myocardial homeostasis for better understanding of late outcome.

## 3. Conclusions

The impact that Kawasaki disease exerts on the myocardium should not be underestimated. Whether from diagnostic or prognostic perspectives, natriuretic peptides represent promising biomarkers for the management and long term care of KD patients. The growing research involving natriuretic peptides seems to bring additional insight onto the diagnosis and management of this challenging disease.
